# p90RSK modulates inter-and intracellular signaling in kidney diseases

**DOI:** 10.3389/fcell.2025.1593914

**Published:** 2025-06-05

**Authors:** Ling Lin, Kebin Hu

**Affiliations:** Department of Medicine and Department of Cell and Biological Systems, The Pennsylvania State University College of Medicine, Hershey, PA, United States

**Keywords:** p90RSK, intracellular signaling, intercellular signaling, extracellular vesicles, kidney diseases

## Abstract

The 90 kDa ribosomal s6 kinases (RSKs) are a group of serine/threonine kinases consisting of 4 RSK isoforms (RSK1-4), of which RSK1 is also named as p90RSK. p90RSK is directly phosphorylated and activated by its immediate upstream mediator extracellular signal-regulated kinase (Erk1/2), followed by activating various signaling pathways through phosphorylating selective downstream substrates. Aberrant induction of p90RSK has been reported in various human diseases including kidney disease suggesting a pathogenic role of p90RSK in these diseases. In response to pathogenic cues, p90RSK not only mediates intracellular signal events leading to cell-specific phenotypes but also modulates intercellular communication impacting the adjacent cellular responses. In this review, we provide an update on the current knowledge regarding the roles of p90RSK-mediated intercellular and intracellular signaling in the pathogenesis and progression of kidney diseases.

## 1 Introduction

The 90 kDa ribosomal s6 kinases (RSKs) are a group of serine/threonine kinases that were initially found in *Xenopus* to be responsible for phosphorylating ribosomal protein S6 (Erikson and Maller, 1985). There are 4 isoforms in the RSK family, including RSK1, RSK2, RSK3 and RSK4 respectively. RSK1 is also designated as p90RSK. RSK1-3 have similar expression patterns and abundance in adult tissues such as heart, brain, lung, kidney, and pancreas (Zeniou et al., 2002). In contrast, expression of RSK4 takes place during development and its deletions are common in x-linked intellectual disability (Yntema et al., 1999). RSKs play important roles in the Ras-mitogen-activated protein kinase (MAPK) signaling cascade and are the direct downstream effectors of extracellular signal-regulated kinase (Erk1/2). Erk1/2 activation directly phosphorylates and activates RSKs (Anjum and Blenis, 2008; Carriere et al., 2008a), which, in turn, activate various intracellular signaling events through selection of different phosphorylation substrates to modulate diverse cellular processes (Lin et al., 2019a), such as cell proliferation, survival, and motility, and/or mediate intercellular signaling relays to regulate the phenotypes of other cells.

## 2 Structure and activation

**FIGURE 1:**
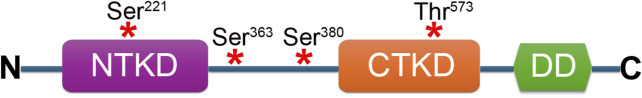
Structural illustration of p90RSK. p90RSK contains two kinase domains: the N terminal kinase domain (NTKD) and the C terminal kinase domain (CTKD), as well as an ERK1/2 docking domain (DD) facilitating their activation by ERK1/2 and four conserved phosphorylation sites including Ser^221^, Ser^363^, Ser^380^ and Thr^573^.

All RSKs share a similar structure with around 75% of which being identical. The crystal structure of human p90RSK was recently released (Muniz et al., 2009). These RSKs contain two functionally diverse domains: the N terminal kinase domain (NTKD) and the C terminal kinase domain (CTKD). The NTKD is part of the kinase AGC family, while the CTKD belongs to the calcium calmodulin dependent kinase (CaMK) family. Functionally, CTKD receives signals from ERK1/2 to auto-phosphorylate RSK, which is important to activate NTKD. Upon activation, NTKD will phosphorylate downstream substrates (Bjorbaek et al., 1995). The CTKD and NTKD domains are bridged by a linker region about approximately 100 amino acids containing regulatory elements (Fisher and Blenis, 1996). Of note, all RSKs contain an ERK1/2 docking domain facilitating their activation by ERK1/2 (MacKenzie et al., 2000). An adjacent location is also important for RSK autophosphorylation, which may play a role in ERK1/2 dissociation and subsequently RSK signal relay (Roux et al., 2003) (Figure 1).

Human RSKs have four conserved phosphorylation sites: Ser^221^, Ser^363^, Ser^380^ and Thr^573^ (Dalby et al., 1998) (Figure 1). The mechanisms of RSK activation are phosphorylation site dependent. Ser^221^ in the NTKD is phosphorylated by phosphoinositide-dependent kinase-1 (PDK1), a constitutively active serine threonine kinase (Jensen et al., 1999). Ser^363^ and Ser^380^ are both located in the linker region between the two kinase domains. Ser^363^ is activated by ERK 1/2 phosphorylation, while Ser^380^ is phosphorylated by CTKD (Vik and Ryder, 1997). Notably, Ser^380^, when phosphorylated, also serves as a docking site for PDK1, which in turn activates Ser^221^ (Frodin et al., 2000). Thr^573^ in the CTKD is also phosphorylated by ERK1/2 (Sutherland et al., 1993). Additionally, RSKs are also regulated by p38 MAPK and fibroblast growth factor receptor-3 (FGFR3). p38 MAPK has been shown to activate RSK in dendritic cells via CTKD activated by MAPK-activated kinases M2 and M3 (Zaru et al., 2007). FGFR3 can interact with RSK2 through tyrosine phosphorylation, which induces its activation by enhancing ERK binding (Kang et al., 2007).

## 3 Downstream substrates

RSKs regulate diverse cellular processes through phosphorylation of selected downstream substrates from a constantly growing list. Both p90RSK (i.e. RSK1) and RSK2 have been shown to promote cell proliferation and growth (Romeo and Roux, 2011), however, it appears that they regulate distinct transcription programs of cell proliferation and growth (Yang et al., 2022). p90RSK phosphorylates and inhibits GSK3β, causing the release of Cyclin D1 and cell proliferation (Lin et al., 2010) and inducing translation initiation factor eIF4B and protein synthesis (Sutherland et al., 1993; Wang et al., 2002). p90RSK phosphorylates Max dimerization protein-1 (Mad1) resulting in release of its suppression of Myc and increased proliferation (Zhu et al., 2008). p90RSK also regulates cell growth and protein synthesis through modulating mTOR pathway. It has been shown to modulate mTOR by phosphorylating both tuberous sclerosis complex 2 (TSC2) and Raptor (Roux et al., 2004; Carriere et al., 2008b). Additionally, RSKs have been shown to interact with c-Fos, an important transcription factor in cell cycle G1 phase (Chen et al., 1993); and phosphorylate p27^kip1^ to induce cell cycle G1 phase progression (Sutherland et al., 1993; Fujita et al., 2003).

p90RSK also plays an important role in cell survival, as it has been shown to phosphorylate Bad to decrease apoptosis, and phosphorylate tumor suppressor death-associated protein kinase (DAPK) to cause its inactivation (Bonni et al., 1999; Anjum et al., 2005; Hu et al., 2008). p90RSK has been shown to influence inflammation through phosphorylating NF-κB inhibitors, IκBα and IκBβ (Schouten et al., 1997; Ghoda et al., 1997; Xu et al., 2006), and through phosphorylating p38 MAPK to induce M1 macrophage survival (Lin et al., 2015). Moreover, p90RSK phosphorylates downstream substrates filamin A and phosphorylating SH3 domain-containing protein (SH3P2) to induce cell motility and migration (Woo et al., 2004; Tanimura et al., 2011). Activated p90RSK phosphorylates Thr^368^ of sentrin/SUMO-specific protease 2 (SENP2), induces SENP2 nuclear export, and reduces the SENP2 activity, which then increases nuclear ERK5 and p53 SUMOylation, leading to endothelial cell (EC) apoptosis and inflammation (Heo et al., 2015; Le et al., 2017; Abe et al., 2017). In a model of diabetic heart disease, activated p90RSK induces ERK5 Ser^496^ phosphorylation, inhibits the association of ERK5 and CHIP ubiquitin ligase by binding to ERK5, which decreases the CHIP ubiquitin ligase activity, suppresses inducible cAMP early repressor (ICER) ubiquitination and degradation, and finally promotes cardiac apoptosis (Le et al., 2012). Activated p90RSK also increases NF-kB activation, VCAM-1 expression, and EC apoptosis through phosphorylating ERK5 Ser^496^ and regulating its transcriptional activity (Vu et al., 2018). Recently, p90RSK has been shown to bind and phosphorylate the E3 ubiquitin ligase MDM2, which increases the stability of MDM2 leading to its binding and ubiquitinating p53 and cell survival (Maietta et al., 2022).

## 4 Intercellular p90RSK signaling and kidney disease

**FIGURE 2:**
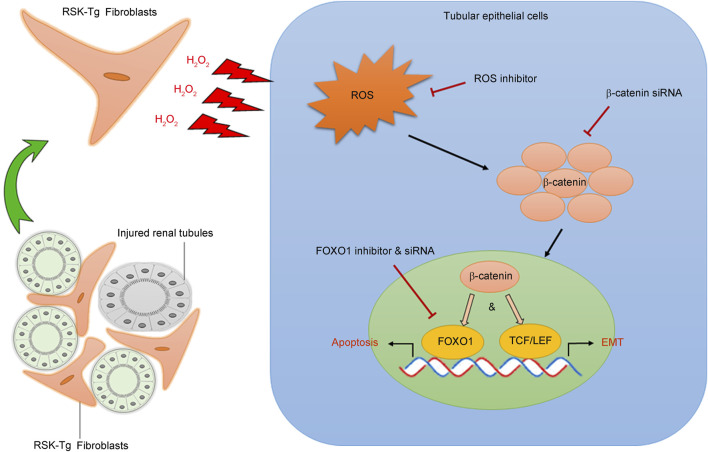
p90RSK modulates intercellular communication between interstitial fibroblasts and tubular epithelial cells. p90RSK-overexpressing fibroblasts produce and release excessive H_2_O_2_ into the surrounding microenvironment causing substantial oxidative stress in the adjacent tubular epithelial cells. Increased epithelial ROS induces β-catenin accumulation and nuclear translocation, which not only activates FOXO1-mediated epithelial apoptosis but also initiates TCF/LET-mediated epithelial transdifferentiation (EMT), leading to the disruption of kidney structure and massive formation of fibrotic scars. Picture was modified from our previous publication (Lin et al., 2019b).

Normal kidney structure and environment depend on epithelial integrity and interactions between epithelial cells and other kidney cells. Obstructive nephropathy (ON) is the major cause of chronic kidney disease (CKD) leading to renal failure in children (Roth et al., 2002) and also happens in adults. However, surgical correction of renal ureteral obstruction does not stop CKD progressing to renal failure in ON patients (Warshaw et al., 1982; McLeod et al., 2018), suggesting a pathogenic role of intrinsic pathway in obstruction-induced CKD. Both interstitial fibroblasts and tubular epithelial cells play essential roles in ON pathogenesis and progression. In response to injury, epithelial cells, especially proximal tubular epithelial cells, not only initiate inflammatory response by producing proinflammatory chemokines, but also undergo apoptotic death, leading to kidney parenchymal destruction. Structurally, fibroblasts reside in the renal interstitium surrounding the tubules formed by epithelial cells. This proximity facilitates interstitial fibroblast-epithelial communication and interactions that are fundamental in maintaining the integrity of the kidney structure and environment, as well as fine-regulated process of adaptation to pathogenic cues (El-Achkar and Dagher, 2015; Borges et al., 2013). Our recent work, using a novel fibroblast-specific wildtype p90RSK-transgenic mouse model, has discovered that p90RSK accelerates obstruction-induced renal fibrogenesis by inducing fibroblast-mediated epithelial apoptosis and transdifferentiation through reactive oxygen species (ROS) (Lin et al., 2019b; Shi et al., 2023) and forkhead box class O1 (FOXO1) pathway (Lin et al., 2019b) (Figure 2). Notably, p90RSK-mediated interactions between inflammatory cells and kidney parenchymal cells remain largely unknown, future investigations are needed for these areas.

### 4.1 Fibroblast p90RSK signaling in tubular epithelial apoptosis

Our recent work has found that fibroblast-specific p90RSK induces tubular epithelial apoptosis and promotes kidney fibrosis (Lin et al., 2019b). As shown in Figure 2, p90RSK-overexpressing fibroblasts produce and release excessive H_2_O_2_, causing ROS accumulation and β-catenin nuclear translocation in the surrounding epithelial cells. Nuclear β-catenin interacts with transcription factor FOXO1 to promote tubular epithelial apoptosis, leading to kidney structural destruction and eventually fibrosis. These results illuminate a novel mechanism regarding the pivotal role of p90RSK-mediated fibroblast-epithelial communications in CKD development and progression. The juxtaposition of fibroblasts and epithelium facilitates their interactions. Under physiological condition, interstitium-epithelial communication plays a fundamental role in maintaining the integrity of the kidney structure and environment (El-Achkar and Dagher, 2015; Borges et al., 2013). During wound healing process, renal interstitial cells, including fibroblasts, also provide a supportive environment to promote tubular epithelial regeneration in response to transient injury (Schiessl et al., 2018). However, persistent pathogenic stimuli will cause extensive activation of signaling mediators, such as p90RSK, and disrupt the delicate healing process, resulting in progressive tissue destruction and loss of function. The recent work shows that p90RSK-transgenic (RSK-Tg) fibroblasts, after chronic obstructive injury, acquire substantially enhanced ability to generate sustained H_2_O_2_ that not only induces epithelial injury, but also further triggers the activation of p90RSK (Sauer et al., 2001; Takeishi et al., 1999) and forms a vicious loop of amplification.

FOXO1, a member of FOXO transcription factor family, controls multiple cellular processes including cell cycle and survival (Lam et al., 2013; Eijkelenboom and Burgering, 2013). We have found that obstruction-induced FOXO1 mediates ROS/β-catenin-induced epithelial apoptosis. β-catenin directly binds to the C-terminal of FOXO1 through its armadillo repeats 1 to 8 and activates FOXO1 transcriptional activity (Essers et al., 2005), which subsequently promotes apoptosis by activating pro-apoptotic proteins including BIM and BAD (Zhang et al., 2011). In general, epithelial apoptosis is followed by regeneration. However, sustained injuries trigger persistent activation of fibroblast p90RSK, which forms a detrimental environment against tubular regeneration and differentiation, resulting in structural destruction and fibrotic scar formation. Thus, it is likely that p90RSK-mediated fibroblast-epithelial communication plays a decisive role in driving kidney fibrosis.

### 4.2 Fibroblast p90RSK signaling in tubular epithelial transdifferentiation

In normal kidney, tubules formed by epithelial cells are surrounded by interstitial fibroblasts, supporting epithelial integrity and functions. In response to chronic pathogenic cues, such as oxidative stress, tubular epithelial cells not only undergo apoptotic death, leading to kidney parenchymal destruction, but also contribute to the population of active fibroblasts through a transdifferentiation process known as epithelial-mesenchymal transition (EMT). EMT, is a reversible process involving loss of epithelial integrity indicated by loss of Ecadherin and gain of contractility and mobility by induction of mesenchymal markers including αSMA, that transiently changes epithelial cells into active fibroblasts, i.e. myofibroblasts, with significantly enhanced matrix-producing capability (Dongre and Weinberg, 2019; Nieto et al., 2016; Acloque et al., 2009). EMT not only plays a fundamental role in embryogenesis and tissue morphogenesis during development, but also is an essential cellular process in wound healing in adults (Dongre and Weinberg, 2019). When the injury is transient, renal interstitial cells, such as fibroblasts, promote epithelial regeneration to proceed wound healing process (Schiessl et al., 2018), while epithelial cells further augment the above process by contributing to the population of active fibroblasts with enhanced matrix-producing capability through EMT to help repair and remodeling. However, when pathogenic damage persists, the finely regulated and delicate healing process will be disrupted, leading to progressive tissue destruction and loss of function. Our most recent work demonstrates that p90RSK-overexpressing fibroblasts produce excessive H_2_O_2_, causing accumulation of ROS and β-catenin in the surrounding epithelial cells. These β-catenins trigger aberrant epithelial transdifferentiation or EMT through inducing TCF/LET-mediated gene expression. These active fibroblasts derived through EMT process, in turn, produce excessive matrix resulting in diffused scaring formation and renal dysfunction (Figure 2). These studies also highlight the significant role of H_2_O_2_ in mediating intercellular p90RSK signaling. Excessive oxidative stress not only induces epithelial β-catenin accumulation and nuclear translocation (Lin et al., 2019b) causing epithelial damage, but also further activates p90RSK (Sauer et al., 2001; Takeishi et al., 1999), forming a vicious cycle to amplify renal damage.

### 4.3 Extracellular vesicles (EVs) and intercellular p90RSK signaling

EVs are a group of nanosized lipid-bound vesicles derived from various cellular origins and are secreted by all cell types and organisms. Although EVs have been subcategorized into several subgroups based on their biogenesis pathway and size, the most common two subsets are exosomes and microvesicles (MVs, also called as ectosomes or microparticles) (Berumen et al., 2021; Kalluri and McAndrews, 2023). The size of exosomes diameter is between 30–150 nm, while that of MVs’ is usually within the range of 50–1,000 nm. Exosomes, containing various biomolecules such as proteins, RNAs, DNAs, lipids, and metabolites, are formed through an endocytic pathway which results in the formation of intraluminal vesicles by inward budding of the multivesicular bodies (Kalluri and McAndrews, 2023). Whereas MVs are formed through outward budding of plasma membrane (Berumen et al., 2021). EVs contain various cell type-specific cargos that play a key role in cell-cell communication.

**FIGURE 3:**
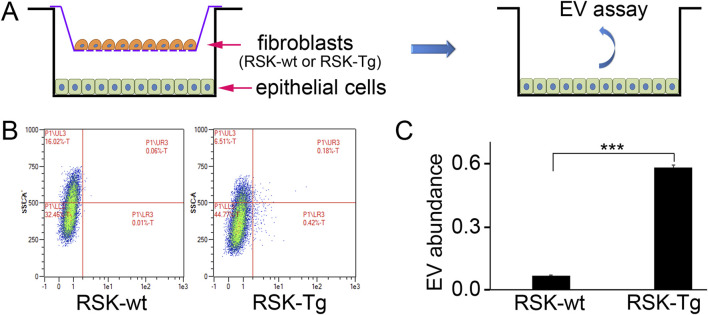
EVs modulate p90RSK-mediated fibroblast-epithelial communication. **(A)** Primary mouse RSK-Tg and RSK-wt fibroblasts were labeled by PKH26 red fluorescence, followed by coculture with mouse TKPT tubular epithelial cells for 72 h. EVs in the epithelial cells were measured and quantified by flow cytometry. **(B)** Representative flow cytometry results. **(C)** Quantification of intraepithelial EV abundance. N = 3, ***P < 0.001.

Our recent works demonstrate an important role of H_2_O_2_ in mediating intercellular p90RSK signaling between interstitial fibroblasts and tubular epithelial cells. Given the significant role of EVs in cell-cell communication, we have conducted a pilot study to test whether p90RSK-overexpressing fibroblast-derived EVs could be deliverable into co-cultured tubular epithelial cells. Briefly, primary mouse RSK-Tg fibroblasts, as well as their wildtype counterparts (RSK-wt), were labeled by PKH26 red fluorescence, followed by coculture with mouse TKPT tubular epithelial cells for 72 h. Then epithelial cells were extracted for intracellular EVs assay and quantitation by flow cytometry. There were dramatically increased RSK-Tg fibroblasts-derived EVs in TKPT epithelial cells (Figure 3) confirming that EVs derived from RSK-Tg fibroblasts were delivered into cocultured epithelial cells. Thus, EVs may also play an important role in mediating intercellular p90RSK signaling between interstitial fibroblasts and tubular epithelial cells. Further investigations are warranted to determine the responsible EV cargo mediators in p90RSK-mediated fibroblast-epithelial communication.

## 5 Intracellular p90RSK signaling and kidney disease

p90RSK signaling is activated in a mouse model of unilateral ureter obstruction (UUO), a classic CKD model. After UUO injury, immune staining shows that phosphorylation of p90RSK and its upstream signaling such as Erk1/2 is markedly induced in the damaged kidneys. The activation of p90RSK, i.e. phosphorylation of p90RSK, correlates with the severity of kidney fibrosis as indicated by induction of matrix proteins and destroyed kidney structure (Lin et al., 2015). *In vitro*, kidney cells treated with profibrotic factors, such as tPA and TGF-beta, display increased p90RSK phosphorylation and activation (Hu et al., 2008; Lin et al., 2015; Das et al., 2010). Collectively, these findings directly point to an important role of p90RSK in the initiation and progression of CKD. After obstructive damage, p90RSK is primarily activated in the interstitium of kidney, where abundant resident fibroblasts reside, and plenty infiltrated inflammatory cells accumulate. p90RSK signaling activated within these cells modulates various cellular processes, such as cell death and proliferation, to contribute to the pathogenesis and progression of CKD.

### 5.1 Kidney fibrosis

**FIGURE 4:**
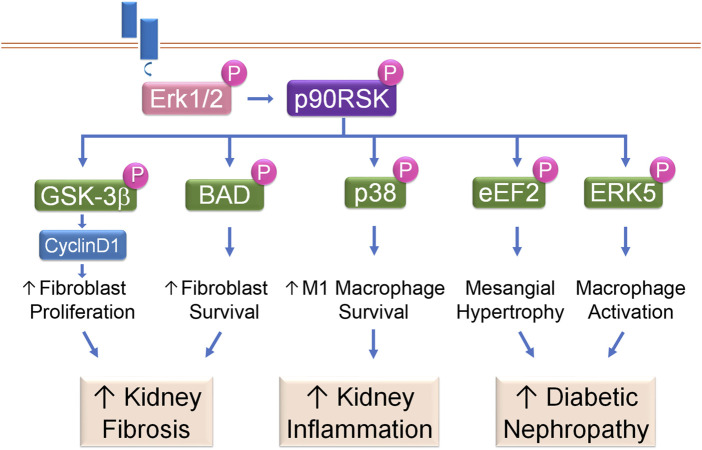
p90RSK modulates intracellular signaling in kidney disease. Receptor-mediated Erk1/2 activation leads to phosphorylation and activation of p90RSK, which then phosphorylates downstream substrates to initiate various cellular processes and the resultant kidney diseases.

Kidney fibrosis is histologically characterized by excessive renal deposition of matrix proteins. Interstitial fibroblasts, as well as their activated form of myofibroblasts, are the primary matrix-producing cells in the kidney. The number of interstitial fibroblasts and myofibroblasts, which is determined by the balance between proliferation and cell death, closely correlates with the severity of tubulointerstitial fibrosis and concomitant decline of kidney function (Hu et al., 2008). We have shown that p90RSK modulates renal fibroblast intracellular signaling to promote renal fibrosis through tilting the balance from cell death to growth to generate substantial amount of kidney interstitial fibroblasts (Lin et al., 2010; Hu et al., 2008). tPA, after binding to its receptor LRP-1 in renal fibroblasts, induces LRP-1 Tyr^4507^ phosphorylation and subsequently activates its downstream Erk1/2 and p90RSK pathway. The activated p90RSK, in turn, phosphorylates its downstream substrates, such as GSK-3β and Bad, to induce fibroblast proliferation and survival (Lin et al., 2010; Hu et al., 2008). GSK-3β exists in a constitutively active form and inhibits cyclinD1 and other downstream mediators through ubiquitination and proteasomal degradation. p90RSK phosphorylates and inactivates GSK-3β, resulting in cyclin D1 stabilization and accumulation, which facilitates fibroblasts entry into the cell cycle S phase and induces cell proliferation (Lin et al., 2010). Bad is a pro-death member of the Bcl-2 protein family. As a downstream substrate of p90RSK, Bad phosphorylation by p90RSK leads to its inactivation and blockage of its entrance into mitochondria after apoptotic injury, and suppression of cytochrome C releasing into cytosol. Decreased cytosol cytochrome C reduces the cleavage and activation of the caspases, resulting in decreased cell death and increased cell survival (Hu et al., 2008). Thus, in tPA-mediated profibrotic pathway, p90RSK activation not only promotes fibroblast survival through p90RSK/Bad/cytochrome C pathway; but also induces fibroblast proliferation through p90RSK/GSK-3β/cyclin D1 signaling (Lin et al., 2010; Hu et al., 2008), which together lead to fibroblast accumulation in the diseased kidneys resulting in progressive renal fibrosis (Figure 4).

### 5.2 Kidney inflammation

Macrophage plays a critical role in kidney inflammation and its accumulation is one of the histological hallmarks of CKD. In diseased conditions, macrophages, including resident and bone marrow monocyte-derived macrophages (Lazarov et al., 2023), are differentiated into a spectrum between two distinct subsets: classically activated M1 and alternatively activated M2 macrophages. M1 macrophages are usually induced by exposure to IFN-γ or lipopolysaccharide (LPS), after which, these macrophages not only produce nitric oxide or reactive oxygen intermediates to protect against bacteria or viral pathogens but also produce abundant proinflammatory cytokines to initiate immune response, recruit more inflammatory cells and exaggerate damage. In contrast, after exposure to IL4, IL10 or IL13, M2 macrophages help to resolve inflammation and promote wound healing and tissue repair (Lin et al., 2015; Arango and Descoteaux, 2014; Mosser, 2003). Resting macrophages (M0) have a finite lifespan but become resistant to apoptosis in response to injury. Our previous work has shown that p90RSK, after phosphorylated and activated by tPA, protects macrophage against apoptosis induced by hydrogen peroxide (H_2_O_2_) or staurosporine indicating that p90RSK mediates damage-triggered apoptosis resistance. Intriguingly, activated p90RSK only protects resting M0 macrophages and classically activated M1 macrophages, but not the alternative activated M2 macrophages, against H_2_O_2_ or staurosporine-induced apoptosis (Lin et al., 2015). In obstruction-induced fibrotic kidneys, increased p90RSK activation correlates to the severer disease condition such as increased extracellular matrix (ECM) accumulation, enhanced M1 macrophage infiltration and more profound proinflammatory cytokine expression (Lin et al., 2015; Lin et al., 2012). There are three known MAPK members: Erk/12, the C-jun N-terminal kinases (JNKs), and the p38 (Raman et al., 2007). It is known that p38 functions parallel to Erk1/2 MAPK, and usually promotes apoptosis (Thornton and Rincon, 2009). However, we have discovered that p38 is a novel downstream substrate of macrophage intracellular p90RSK mediating its cytoprotective effects (Lin et al., 2015). Thus, p90RSK promotes survival and accumulation of macrophages, especially the M1 macrophages, through phosphorylating its intracellular p38 MAPK pathway. These increased M1 macrophages, in turn, produce a panoply of proinflammatory cytokines and chemokines, resulting in increased inflammatory cell recruitment, severer inflammatory response, and excessive renal damage (Figure 4).

### 5.3 Diabetic nephropathy (DN)

DN is histologically characterized by thickened tubular basal (TBM) and glomerular basement (GBM) membranes, excessive ECM deposition and progressive mesangial hypertrophy. DN is one of the leading causes of end stage renal disease (Ni et al., 2015). Around 30%–40% of the total glomerular cell population are mesangial cells, which play an important role in maintaining glomerular integrity (Scindia et al., 2010). Mesangial hypertrophy is characterized by excessive mesangial matrix deposition, which eventually leads to glomerulosclerosis, if not under control. TGF-β is the most prominent growth factor mediating the hypertrophic effect of hyperglycemia in DN (Mahimainathan et al., 2006). TGF-β has been shown to induce Erk1/2 and p90RSK activation in mesangial cells. Activated p90RSK phosphorylates and inactivates eukaryotic elongation factor2 (eEF2) kinase, leading to decreased eEF2 phosphorylation and augmented activity. Activation of eEF2 promotes matrix production and deposition in the mesangial region causing hypertrophy. Mesangial intracellular p90RSK is indispensable to TGF-β-induced mesangial hypertrophy because dominant-negative p90RSK abolishes the effects of TGF-β (Das et al., 2010) (Figure 4).

Oxidative stress plays an important role in DN pathogenesis and progression. In monocytes/macrophages treated with various combination antiretroviral therapies (cARTs), p90RSK is activated, which then phosphorylates Ser^496^ of ERK5, inhibits NRF2-ARE activity, reduces the telomere length and decreases antioxidant expression, resulting in increased sensitivity of monocytes/macrophages to oxidative stress. Activated macrophage intracellular p90RSK signaling cascade also induces the expression of pro-inflammatory genes such as TNFα, and decreases the expression of efferocytosis-related genes, such as Gas6, causing inflammation, matrix deposition, and sclerosis (Singh et al., 2018) (Figure 4).

### 5.4 Glomerular diseases

Most glomerular diseases are presented with proteinuria. In physiological condition, the healthy glomerular filtration barrier, consisting of endothelium, GBM, and podocytes, only allows the passthrough of metabolic wastes but not proteins larger than albumin from plasma inside the glomerular capillaries into resultant urine within Bowman’s capsule. Podocytes cover the outer surface of the GBM, and their long-interdigitated foot processes form filtration slits and are critical for the integrity of glomerular filtration barrier. As terminally differentiated cells, podocytes cannot regenerate when injured. In a puromycin aminonucleoside (PAN)-induced injury model, podocytes undergo apoptosis and detach from GBM, leading to glomerular filtration barrier integrity disruption and proteinuria (Zhao et al., 2018). It has been found that calcimimetic R-568 induces Erk1/2-mediated p90RSK/CREB signaling cascade, alleviates PAN-induced proteinuria, attenuates glomerulosclerosis, and improves GFR. p90RSK has been shown to mediate protective effect of R568 through activating the pro-survival signaling of Bad and Bcl-xl and suppressing PAN-induced podocyte apoptosis and damage (Oh et al., 2011).

### 5.5 Other kidney diseases

Epithelial intracellular p90RSK signaling has also been implicated in other kidney diseases including hypocitraturia, kidney stone, virus-induced kidney injury, as well as renal cell carcinoma (RCC). p90RSK has been shown to mediate IL11-induced tubular epithelial dedifferentiation (Widjaja et al., 2022). In a mouse model of bone metastasis of RCC, intracardiac injection of calcium-sensing receptor (CaSR)-transfected RCC cells demonstrates an increased rate of bone metastasis. Calcium-induced SHC, Akt, Erk, p90RSK and JNK in CaSR-transfected RCC cells have been associated with enhanced adhesion to endothelial cells and ECM components, as well as calcium-induced chemotactic cell migration and proliferation (Frees et al., 2018). Intriguingly, although p90RSK signaling is activated in a genetic PKD1 inaction model of polycystic kidney disease (PKD), p90RSK inhibition has little effect on the cyst growth (Shibazaki et al., 2008). Thus, the roles of intracellular p90RSK signaling in some kidney diseases are context dependent.

Acidified media has been shown to activate the citrate transporter NaDC-1 through Raf1, ERK1/2 and p90RSK signaling in the opossum kidney proximal tubule cells, suggesting an important role of p90RSK signaling in hypocitraturia and kidney stone formation (Zacchia et al., 2018). It has also been found that severe acute respiratory syndrome (SARS)-coronavirus (CoV) infection induces p38 MAPK-mediated Ser^380^ phosphorylation of p90RSK but not through Erk1/2-induced Thr^573^ phosphorylation in kidney epithelial cells, indicating a role of epithelial intracellular p90RSK signaling in virus-induced kidney damage (Mizutani et al., 2006). During the recovery phase after acute kidney injury, surviving epithelial cells de-differentiate, migrate to the injury site, proliferate and then re-differentiate to establish epithelial polarity and restore kidney function (Susnik et al., 2015). HGF, PMA and EGF-induced p90RSK signaling is implied in the motility response of kidney epithelial cells (Tanimura et al., 1998), indicating a critical role of epithelial intracellular p90RSK signaling in mediating kidney repair and regeneration after acute injury. Most recently, activated epithelial intracellular p90RSK has been shown to mediate anisodamine-induced renal recovery after acute ischemia/reperfusion injury (Zhang et al., 2021).

## 6 Conclusion and future perspectives

Emerging evidences support an essential role of p90RSK in mediating a complex intercellular and intracellular signaling network to modulate diverse cellular processes to initiate various progressive kidney diseases. Notably, the roles of p90RSK in kidney diseases are context dependent, because its induction is an initial finely regulated wound-healing response until the sustained damage-caused chaotic signal and cellular reactions leading to aberrant activation of p90RSK signal cascades and subsequent tissue destruction and scar formation. Currently, the role of p90RSK in kidney disease remains largely unknown. Future investigations should not only further clarify its renal pathogenic roles but also focus on its roles in mediating interactions between inflammatory cells and renal parenchymal cells during CKD pathogenesis and progression, as well as the development of p90RSK-specific treatment.
